# Morphogenesis of Plasmodium zoites is uncoupled from tensile strength

**DOI:** 10.1111/mmi.12297

**Published:** 2013-07-05

**Authors:** Annie Z Tremp, Victoria Carter, Sadia Saeed, Johannes T Dessens

**Affiliations:** Department of Pathogen Molecular Biology Faculty of Infectious and Tropical Diseases, London School of Hygiene & Tropical MedicineKeppel Street, London, WC1E 7HT, UK

## Abstract

A shared feature of the motile stages (zoites) of malaria parasites is a cortical cytoskeletal structure termed subpellicular network (SPN), thought to define and maintain cell shape. *Plasmodium* alveolins comprise structural components of the SPN, and alveolin gene knockout causes morphological abnormalities that coincide with markedly reduced tensile strength of the affected zoites, indicating the alveolins are prime cell shape determinants. Here, we characterize a novel SPN protein of *Plasmodium berghei* ookinetes and sporozoites named G2 (glycine at position 2), which is structurally unrelated to alveolins. G2 knockout abolishes parasite transmission and causes zoite malformations and motility defects similar to those observed in alveolin null mutants. Unlike alveolins, however, G2 contributes little to tensile strength, arguing against a cause-effect relationship between tensile strength and cell shape. We also show that G2 null mutant sporozoites display an abnormal arrangement of their subpellicular microtubules. These results provide important new understanding of the factors that determine zoite morphogenesis, as well as the potential roles of the cortical cytoskeleton in gliding motility.

## Introduction

Morphogenesis is the biological process that causes an organism to develop its shape, and forms a key aspect of developmental biology. *Plasmodium* species, the causative agents of malaria, possess three invasive and motile life stages (zoites): the merozoite, the ookinete and the sporozoite. Morphogenesis of these three zoite species is likely to follow similar events, because despite their very different sizes and shapes they have as a common feature a unique cortical cytoskeletal structure known as the pellicle. The pellicle is composed of the plasma membrane and an underlying double membrane structure named inner membrane complex (IMC) (Bannister *et al*., 2000; Morrissette and Sibley, 2002a; Santos *et al*., 2009). The IMC is equivalent to a system of flattened vesicles known as alveoli, which are a unifying morphological feature linking the Apicomplexa with dinoflagellates and ciliates to create the protist infrakingdom Alveolata. The IMC is supported on its cytoplasmic side by a network of intermediate filaments (IF) named the subpellicular network (SPN), which forms an internal cytoskeletal basket that supports the pellicular membranes and provides mechanical strength to the cell (Mann and Beckers, 2001). The SPN effectively separates the main cytosol from a smaller cortical cytoplasm, which contains the molecular machinery (glideosome) that drives apicomplexan parasite motility, invasion and egress (Kappe *et al*., 2004; Keeley and Soldati, 2004; Sibley, 2004; Soldati and Meissner, 2004; Baum *et al*., 2006; Matuschewski and Schuler, 2008; Santos *et al*., 2009). Completing the cortical cytoskeletal structure of the zoites are subpellicular microtubules that originate at the zoite's apical ring structures, and that run down the cell along the cytoplasmic side of the IMC (Bannister *et al*., 2000; Morrissette and Sibley, 2002a; Santos *et al*., 2009). The number of subpellicular microtubules varies both between parasite species and between their life stages. *Plasmodium* merozoites in particular possess a limited repertoire of subpellicular microtubules (in *Plasmodium falciparum* these are termed *falciparum* merozoite-associated assemblage of subpellicular microtubules, or *f*-MAST) compared with ookinetes and sporozoites (Morrissette and Sibley, 2002a), possibly reflecting the merozoites' relatively limited motility requirements.

The IMC1 proteins form an Apicomplexa-specific family of IF proteins that comprise core components of the SPN (Mann and Beckers, 2001; Khater *et al*., 2004). The IMC1 proteins are characterized by possessing one or more domains distantly related to articulins, cytoskeleton proteins of free-living protists. Structurally related proteins from dinoflagellate algae and ciliates have recently been added to this protein family renamed ‘alveolins’, which now define the Alveolata infrakingdom (Gould *et al*., 2008). We have previously shown in *Plasmodium berghei* that disruption of individual alveolin family members expressed in sporozoites (IMC1a), in ookinetes (IMC1b), or in both these zoites (IMC1h) leads to similar morphological abnormalities of the zoite stages in which they are expressed (Khater *et al*., 2004; Tremp *et al*., 2008; Tremp and Dessens, 2011), identifying a clear role for the alveolins in zoite morphogenesis. These morphological abnormalities are characterized by a reduction in cell length, as well as the presence of a bulging area typically located near the centre of the cell and associated with the position of the nucleus (Khater *et al*., 2004; Tremp *et al*., 2008; Tremp and Dessens, 2011; Volkmann *et al*., 2012). In all alveolin null mutants studied, these malformations are accompanied by a marked reduction in tensile strength of the affected zoites (Khater *et al*., 2004; Tremp *et al*., 2008; Tremp and Dessens, 2011). This, in turn, identified the provision of tensile strength as a likely mechanism by which the alveolins facilitate zoite morphogenesis and help maintain cell shape.

In this study we identify and describe a new *Plasmodium* protein, named G2 (glycine at position 2) that has no structural orthologues outside the phylum Apicomplexa, and has no structural paralogues within it. We provide evidence that G2 is lipid-modified through myristoylation, localizes to the pellicle of ookinetes and sporozoites, and behaves similarly to alveolins with respect to its loss-of-function phenotypes. A notable exception to this is that – unlike the alveolins – G2 plays only a minor role in providing tensile strength to the zoites. Thus, G2 plays an essential role in morphogenesis without influencing tensile strength, in contrast to the alveolins, which affect both properties.

## Results

### Identification and structure of G2

Many *Plasmodium* alveolins are predicted to be translationally repressed in female gametocytes of *P. berghei* (Mair *et al*., 2006). Translational repression is a process of translational silencing of mRNA that is specific to female gametocytes and involved in development of the parasite post-fertilization (Mair *et al*., 2006). In addition, many apicomplexan alveolins are predicted to be post-translationally acylated, reflected by the presence of conserved amino- and/or carboxy-terminal cysteine motifs (Mann and Beckers, 2001; Khater *et al*., 2004; Anderson-White *et al*., 2011). When we applied these two screens to the available *P. berghei* gene models we identified a conserved protein with unknown function here named G2 (PBANKA_083040). This protein is orthologous to the recently identified ILP1 of *Toxoplasma* (TGME49_313380) (Lorestani *et al*., 2012). The screen also identified two new *Plasmodium* IMC1 protein/alveolin family members: PBANKA_070710 and PBANKA_112040. Transcript levels of *g2* in *P. berghei* are over 7.5 times higher in wild-type gametocytes than in gametocytes of null mutants for the DDX6-class RNA helicase DOZI (development of zygote inhibited) (Mair *et al*., 2006), which is a strong indicator of translational repression.

**Figure 1:**
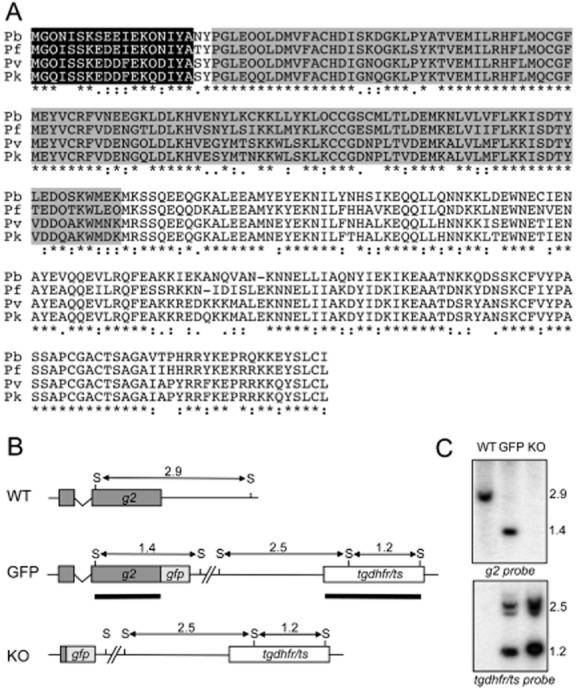
Sequence, structure and genetic modification of *P. berghei* G2.A. Multiple amino acid sequence alignment of the predicted G2 proteins from *P. berghei* (Pb), *P. falciparum* (Pf), *P. vivax* (Pv) and *P. knowlesi* (Pk). Conserved amino acid identities (asterisks) and similarities (colons and points) are indicated underneath. Highlighted are the predicted N-myristoylation motif (black) and calcium-binding domain (grey). The alignment was generated with clustalw. N-myristoylation was predicted using http://mendel.imp.ac.at/myristate/SUPLpredictor.htm and calcium binding was predicted using http://supfam.cs.bris.ac.uk/SUPERFAMILY.B. Schematic diagram of wild-type (WT) and genetically modified *g2* loci on genomic DNA. Indicated are positions of SphI restriction sites (S), and expected SphI restriction fragments (horizontal arrows) with approximate sizes shown in kb. Sequences corresponding to the probes used are indicated by thick lines.C. Southern blot of SphI-digested parasite genomic DNA using probes specific to *g2* and *tgdhfr/ts* sequences.

The predicted *P. berghei* G2 protein is encoded by two exons and is composed of 272 amino acids with a calculated M_r_ of 31 728. Homology searches reveal that it is highly conserved among *Plasmodium* orthologues (> 75% amino acid identity) (Fig. 1A). G2 is predicted to be lipid modified on the glycine residue at position 2 through myristoylation, reflected by the presence of a strong canonical amino-terminal myristoylation motif (Maurer-Stroh *et al*., 2002) (Fig. 1A). In addition, a putative calcium-binding domain related to the EF-hand superfamily (de Lima Morais *et al*., 2011) is predicted to lie downstream of the myristoylation motif with E-values of 1.86 e^−5^ (*Plasmodium knowlesi*), 3.97 e^−5^ (*P. vivax*), 1.45 e^−4^ (*P. falciparum*) and 4.32 e^−4^ (*P. berghei*). Predicted G2 orthologues were identified in *Toxoplasma* (e.g. TGME49_313380), *Cryptosporidium* (e.g. EAK88688.1), *Babesia* (e.g. EDO06546.1) and *Theileria* (e.g. EAN32844.1) (Supplementary material Fig. S1), but not in organisms outside the Apicomplexa.

### Generation and molecular analyses of transgenic parasite lines

To study expression and localization of G2 we generated a transgenic *P. berghei* line by double-crossover homologous recombination (Waters *et al*., 1997) that expresses full-length G2 fused to a carboxy-terminal enhanced GFP tag (Fig. 1B). To study the function of G2 and its contribution to parasite development we generated another transgenic parasite, which is depleted of the *g2* coding sequence. In this parasite line GFP is left as a reporter gene under control of the endogenous *g2* promoter (Fig. 1B). After transfection of purified schizont preparations, pyrimethamine-resistant parasites were selected and dilution cloned. Diagnostic PCR across the predicted integration sites showed correct integration of the *tgdhfr* cassette into the *g2* locus as well as absence of the unmodified *g2* allele (data not shown). This was confirmed by Southern analysis of SphI-digested genomic DNA (Fig. 1C). Hybridization with a *g2*-specific probe gave rise to a 2.9 kb band in the parental wild-type (WT) parasites; a 1.4 kb band in G2/GFP parasites; and no signal in G2-KO parasites, as predicted (Fig. 1C). A *tgdhfr/ts*-specific probe gave rise to specific signals of the expected sizes in the G2/GFP and G2-KO parasites, but not in WT parasites (Fig. 1C). These combined results confirmed correct integration of the recombinant *g2* and *tgdhfr/ts* alleles into the *g2* locus.

### Life stage expression and subcellular localization of G2

**Figure 2:**
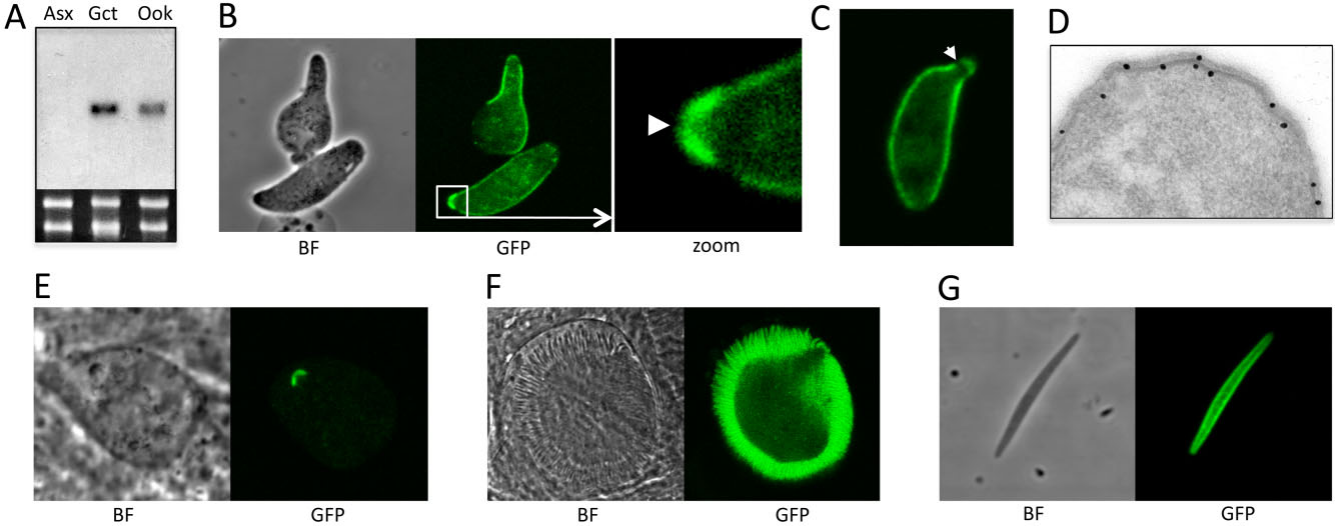
Gene expression and subcellular localization of G2.A. Northern blot of total RNA extracted from purified asexual parasites (Asx), gametocytes (Gct) and ookinetes (Ook). As a loading control is an ethidium bromide-stained gel of total RNA showing the large and small subunit ribosomal RNA species.B. Confocal microscope bright-field (BF) and green fluorescent (GFP) images of a retort (top) and mature ookinete (bottom). The boxed area is enlarged (zoom), with arrowhead pointing to pore in the centre of the cap structure.C. Ookinete with a typical apical extension (protuberance), showing gap (arrow) between the cap-like structure and the pellicle.D. Immunogold labelling (with silver enhancement) of G2 in an ookinete, showing association with the pellicle.E. Confocal microscope images of a young oocyst showing that the pellicle disappears before the cap-like structure.F. Confocal microscope images of a mature, sporulating oocyst.G. Confocal microscope images of a midgut sporozoite.This figure is available in colour online at http://wileyonlinelibrary.com.

Northern analysis of purified asexual blood stages, gametocytes and ookinetes revealed the presence of *g2*-specific mRNA in ookinetes and gametocytes, with highest levels in the latter (Fig. 2A), an observation that is fully consistent with the predicted translational repression in the gametocyte. Expression of the *g2* gene product was studied using a transgenic parasite line expressing a full-length, carboxy-terminally GFP-tagged fusion protein from its endogenous promoter, allowing us to study G2 expression and subcellular localization in live parasites. These parasites developed normally in mice and mosquitoes and were readily transmitted by infected mosquito bites, demonstrating that the GFP fusion to the G2 protein did not adversely affect parasite development. Examination of blood-stage parasites revealed very weak cytoplasmic GFP-based fluorescence in female gametocytes (not shown). Fluorescence levels increased concomitant with ookinete development, culminating in mature ookinetes with strong GFP signal concentrated both at the periphery of the cell and in an apically located cap-like structure with a pore in the centre (Fig. 2B). The inverse relationship between mRNA and protein abundance in gametocytes and ookinetes is typical of translationally repressed genes (e.g. see *P*25 and *P*28) (Paton *et al*., 1993; del Carmen Rodriguez *et al*., 2000; Braks *et al*., 2008). We also observed ookinetes displaying a narrow extension at the apical end with the cap structure at its extremity and seemingly discontinuous with the rest of the cortical structure (Fig. 2C), possibly corresponding to the apical ‘protuberance’ reported in *P. berghei* ookinetes by electron microscopy (Garnham *et al*., 1969). Retorts (i.e. developing, immature ookinetes) displayed cortical fluorescence only in the ‘ookinete’ portion that protrudes from the spherical zygote (Fig. 2B), indicating that G2 associates with the pellicle structure and not the plasma membrane. Indeed, immunogold labelling of ookinete ultrathin sections saw the large majority of gold particles associated with the pellicle (Fig. 2D). The cap-like structure was notably absent in retorts, indicating that it forms during the final stages of ookinete development or maturation.

In young oocysts at 4 days after infecting mosquitoes, GFP fluorescence had largely disappeared, except for the apical cap structure (Fig. 2E), and at 6 days post-infection the majority of oocysts lacked any discernible fluorescence. GFP-based fluorescence was again observed in oocysts from around 8–10 days after infecting mosquitoes, reaching peak levels during sporulation (Fig. 2F). The G2::GFP fusion protein concentrated at the periphery of the sporozoites, but in contrast to ookinetes was found associated with neither anterior nor posterior end (Fig. 2G). This is consistent with the observation that *Plasmodium* sporozoites, contrary to ookinetes, do not possess a cap-like structure (Vanderberg *et al*., 1967).

### Cell morphology of G2 null mutants

**Figure 3:**
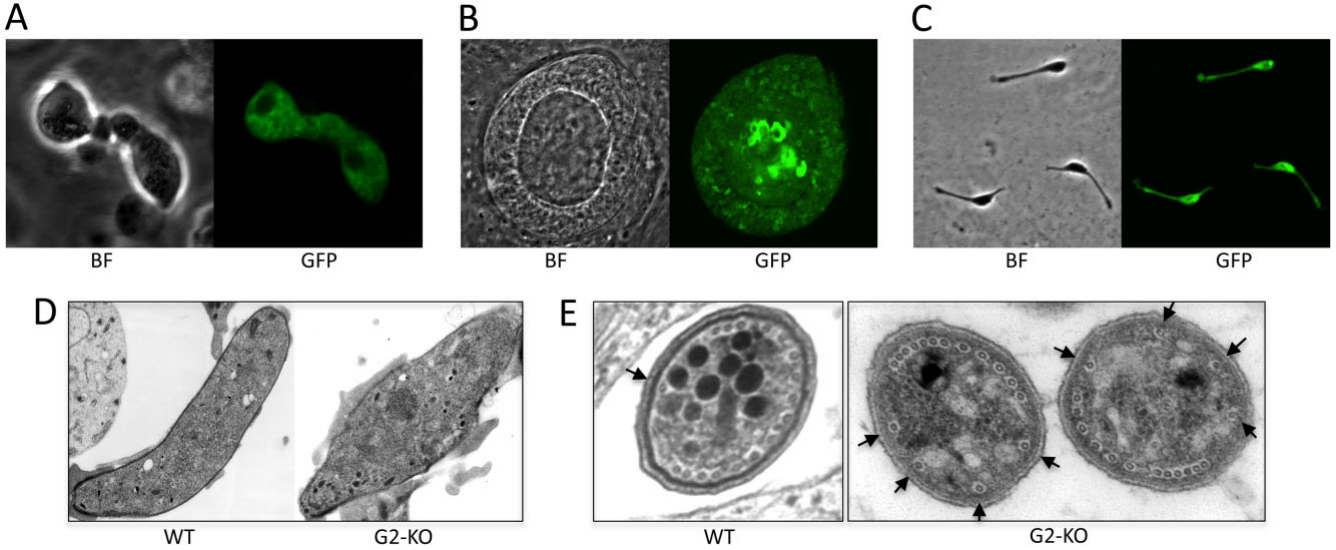
Morphology of G2 null mutant parasites.A. Confocal bright-field (BF) and green fluorescent (GFP) microscope images of two ookinetes.B. Confocal images of a sporulating oocyst.C. Confocal images of three midgut sporozoites.D. Electron micrographs of a wild-type (WT) ookinete (left panel) and a G2-KO ookinete (right panel). The pellicle and collar are still present in the null mutant.E. Electron micrographs of a cross-section through WT and G2-KO midgut sporozoites, showing an abnormal organization of the subpellicular microtubules. The arrows point to the solitary microtubule (WT), or the four microtubules that occupy approximately half of the circumference of the cell (G2-KO).This figure is available in colour online at http://wileyonlinelibrary.com.

The function of G2 and its contribution to parasite development and infectivity were studied using a G2 null mutant (G2-KO) (Fig. 1B). Gametogenesis occurred normally in this parasite line, and ookinetes developed *in vitro* and *in vivo* in numbers comparable to WT and G2/GFP parasites. However, closer examination of G2-KO ookinetes revealed that their morphology was abnormal. Compared with G2/GFP ookinetes that express functional G2 protein, G2-KO ookinetes were typically wider and shorter and possessed a bulging area near the central part of the cell (Fig. 3A). This morphology phenotype in ookinetes is highly similar to that observed in parasite lines that lack the alveolins IMC1b or IMC1h (Tremp *et al*., 2008; Tremp and Dessens, 2011; Volkmann *et al*., 2012), and in direct comparison G2-KO ookinetes were indistinguishable from IMC1b-KO or IMC1h-KO ookinetes.

G2-KO parasite-infected mosquitoes formed oocysts that developed normally and formed large numbers of sporozoites (Fig. 3B). However, the sporozoites were also of abnormal shape being shorter and possessing an enlarged, bulging area typically near the middle or posterior end of the cell (Fig. 3C). This morphology phenotype is highly similar to that of sporozoites that lack alveolins IMC1a or IMC1h (Khater *et al*., 2004; Tremp and Dessens, 2011; Volkmann *et al*., 2012). The same morphological abnormalities were observed in an independent clone of the G2-KO parasite line indicating that this phenotype is the result of the *g2* gene disruption rather than clonal variation.

Examination of the ultrastructure of ookinetes by electron microscopy showed the presence of an apparently normal pellicle structure and apical collar in the G2 null mutants (Fig. 3D). A normal looking pellicle structure was also present in G2 null mutant sporozoites (Fig. 3E). Wild-type *P. berghei* sporozoites typically possess 16–17 subpellicular microtubules that are arranged in an asymmetrical manner in which all but one closely spaced microtubules occupy approximately two-thirds of the perimeter, while a solitary microtubule is positioned opposite (Vanderberg *et al*., 1967) (Fig. 3E). In contrast, the subpellicular microtubule organization in G2 null mutant sporozoites was notably different with not one, but four equally spaced microtubules occupying approximately one half of the circumference, and the remaining closely spaced microtubules in the opposite half (Fig. 3E). These observations indicate that knockout of G2 affects the organization of the subpellicular microtubules.

### Tensile strength of G2 null mutants

**Figure 4:**
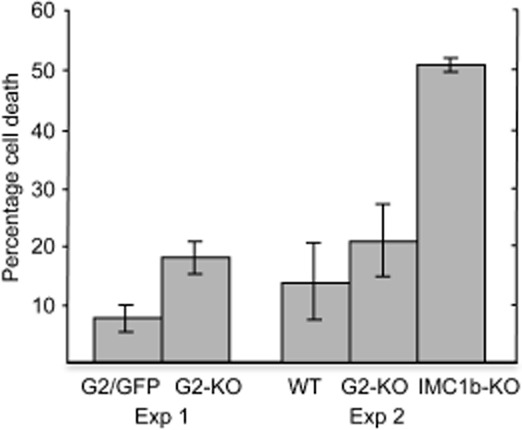
Tensile strength of G2 null mutant ookinetes. Percentage cell death measured after hypo-osmotic shock of ookinetes from parasite lines G2/GFP and G2-KO (Experiment 1), or of wild-type (WT), G2-KO and IMC1b-KO parasites (Experiment 2). Error bars indicate standard deviations from three independent experiments. Values were normalized to 100% viability in untreated cells. At least 100 ookinetes were scored for each sample.

Null mutants of the alveolins IMC1a, IMC1b and IMC1h display markedly reduced tensile strength in the zoite stages in which the proteins are prominent (Khater *et al*., 2004; Tremp *et al*., 2008; Tremp and Dessens, 2011). The similarities in shape of the G2 null mutant ookinetes and sporozoites with those of the corresponding life stages of the alveolin null mutants led us to test whether the tensile strength of our G2 null mutants was also adversely affected. This was assessed by subjecting ookinetes to hypo-osmotic shock: these conditions cause cells to draw in water and swell, and the degree of hypo-osmotic stress a cell can tolerate is a measure of its tensile strength (Menke and Jockusch, 1991). Reproducibly, exposure to hypo-osmotic conditions caused only about 10% more cell death in G2-KO ookinetes as it did in ookinetes expressing functional G2 (Fig. 4), indicating that the protein makes at best only a small contribution to the tensile strength of the zoite. This contrasted with the markedly reduced tensile strength reported for alveolin null mutants. Indeed, direct comparison with IMC1b-KO parasites confirmed that G2 null mutant ookinetes withstand osmotic stress much better than their alveolin null mutant counterparts, of which less than half the cells survived the treatment (Fig. 4).

### Infectivity of G2 null mutants

To assess the infectivity of G2-KO parasites to *Anopheles stephensi* vector mosquitoes, parasite-infected insects were analysed for oocyst development at 10 days post-infection in direct comparison with G2/GFP parasites expressing the functional protein. Reproducibly, oocyst numbers obtained in G2-KO parasites-infected mosquitoes were markedly and significantly reduced compared with those found in mosquitoes infected with G2/GFP parasites (*P* < 0.0001) (Table 1), demonstrating that knockout of G2 expression adversely affects ookinete infectivity. We failed to detect discernible numbers of G2-KO sporozoites in the salivary glands, and consistent with this observation we were unable to transmit this parasite to naïve mice by sporozoite-infected mosquito bites (data not shown). These results indicate that G2 is required for sporozoite infectivity.

**Table 1:** Impact of *g2* gene disruption on *P. berghei* oocyst development in *Anopheles stephensi* mosquitoes

Experiment	Parasite line	Mean/median number of oocysts/mosquito (range)^a^	Prevalence of infection (%)
I	G2/GFP	218/129 (0–934)	93
	G2-KO	2.2/2.0 (0–10)	90
II	G2/GFP	29.4/21.0 (0–87)	95
	G2-KO	0.4/0.0 (0–4)	20

a Thirty and 40 mosquitoes dissected per parasite line for experiments I and II respectively.

### Myristoylation of G2

**Figure 5:**
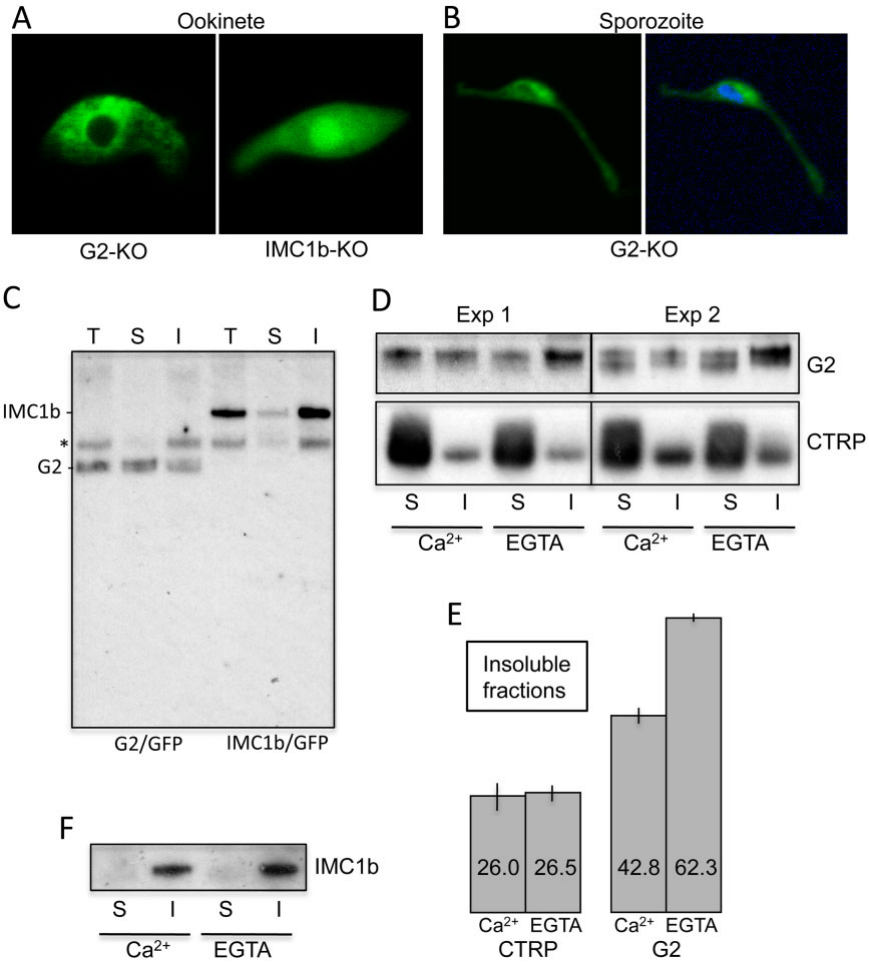
Myristoylation and detergent solubility of G2.A. Confocal green fluorescence images of a typical G2-KO and IMC1b-KO ookinete, clearly showing the differential localization of the GFP reporter protein fused to amino-terminal amino acids of G2 (myristoylated) and IMC1b (non-myristoylated) respectively.B. Confocal green fluorescence image of a G2-KO oocyst. Hoechst DNA co-staining (blue) shows that GFP is mostly absent from the sporozoite nucleus.C. Western blot analysis of the distribution of G2 in parasite line G2/GFP, and IMC1b in parasite line IMC1b/GFP, in deoxycholate-soluble (S) and deoxycholate-insoluble (I) fractions. T = total protein. The asterisk marks the position of an approximately 65 kDa protein that reacts non-specifically with the anti-GFP antibodies (Saeed *et al*., 2012).D. Distribution of G2 and CTRP in deoxycholate-soluble (S) and deoxycholate-insoluble (I) fractions in the presence (Ca^2+^) or absence (EGTA) of calcium.E. Quantification of CTRP and G2 in the deoxycholate-insoluble fractions in the presence or absence of calcium from (D), expressed as relative amounts (%).F. Distribution of IMC1b in deoxycholate-soluble and -insoluble fractions remains unchanged in response to calcium.This figure is available in colour online at http://wileyonlinelibrary.com.

Our gene targeting DNA constructs were designed in such a way that the GFP reporter expressed in the G2 null mutant parasites would be fused to the amino-terminal amino acids of the G2 protein. As this short amino-terminal sequence contains the predicted N-myristoylation recognition signal (Maurer-Stroh *et al*., 2002), it allowed us to assess the potential effects of this lipid modification on the subcellular localization of the reporter protein. A null mutant of IMC1b (Tremp *et al*., 2008) expressing a GFP reporter fused to a short amino-terminal sequence derived from IMC1b (not predicted to posses a myristoylation signal) served as a control in this experiment. Localization of the GFP reporter in G2-KO parasites was markedly different from that of the IMC1b null mutants, most obviously reflected by the exclusion of GFP from the nucleus (Fig. 5A). The same was also observed in sporozoites (Fig. 5B). In addition, the GFP fluorescence in the cytoplasm of G2-KO ookinetes generally had a more uneven, patchy appearance as opposed to the smoothly distributed fluorescence observed in the cytoplasm of the IMC1b knockouts (Fig. 5A), pointing to an increased association of GFP with intracellular membranes. These differences in subcellular GFP distribution in response to N-myristoylation are consistent with those observed in *Toxoplasma gondii* (Beck *et al*., 2010) and in mammalian cells (McCabe and Berthiaume, 1999) and indicate that G2 is genuinely myristoylated in *Plasmodium*. Myristoylated GFP was not targeted to the pellicle, suggesting that myristoylation alone is not sufficient for correct sorting of G2 to the pellicle. The G2 protein also contains multiple predicted palmitoylation sites (CSS-Palm), which could cooperate in pellicle targeting.

### Detergent solubility of G2

It was previously shown in *Toxoplasma* tachyzoites that the SPN is poorly detergent-soluble and is resistant even to extraction with deoxycholate (Mann and Beckers, 2001; Beck *et al*., 2010). To investigate the relative solubility of G2 and the alveolin IMC1b, ookinetes from parasite lines G2/GFP and IMC1b/GFP, respectively, were cultured, purified and subjected to stringent 1% sodium deoxycholate extraction followed by Western blot analysis of equivalent amounts of the soluble and insoluble fractions using anti-GFP antibodies. Similar to other alveolins, IMC1b::GFP was predominantly found in the deoxycholate-insoluble fraction (Fig. 5C), for the first time showing that the SPN of *Plasmodium* ookinetes, too, is poorly detergent-soluble. The G2::GFP fusion protein migrated at approximately 60 kD – its predicted size – as two bands very close in size. In contrast to IMC1b, G2 was more equally distributed in deoxycholate-soluble and -insoluble fractions (Fig. 5C). For comparison, the ookinete-specific transmembrane protein CTRP (circumsporozoite and TRAP-related adhesive protein) (Dessens *et al*., 1999) was principally present in the deoxycholate-soluble fraction, as expected (Fig. 5D). The pellicular localization of G2 combined with its limited deoxycholate-solubility suggests that it is associated with the SPN albeit not as tightly as alveolins, possibly because of having a hydrophobic/lipophilic myristate group attached. Notably, the G2 orthologue in *Toxoplasma*, ILP1, is almost entirely deoxycholate-insoluble despite a more stringent extraction protocol (Lorestani *et al*., 2012). This could reflect the fact that *Tg*ILP1 is not predicted to be N-myristoylated (NMT MYR Predictor).

Because of the putative calcium-binding domain present in G2 we tested the effect of calcium on G2 solubility. In these experiments G2::GFP was reproducibly and significantly less deoxycholate-soluble when Ca^2+^ was removed by chelating with EGTA (*P* < 0.01) (Fig. 5D and E). By comparison, Ca^2+^ chelation had no significant effect on the deoxycholate-solubility of CTRP (Fig. 5D and E) or IMC1b (Fig. 5F), as expected. These results indicate that Ca^2+^ increases the ability of deoxycholate to extract the G2 protein away from the SPN. It should be noted that because of competition of the antibodies for their binding sites (i.e. molecular crowding), the western blot signals are skewed in favour of the fraction in which the target protein is less abundant. Differences in solubility are therefore likely to be more pronounced than the western blots indicate.

### Gliding motility of G2 null mutants

**Figure 6:**
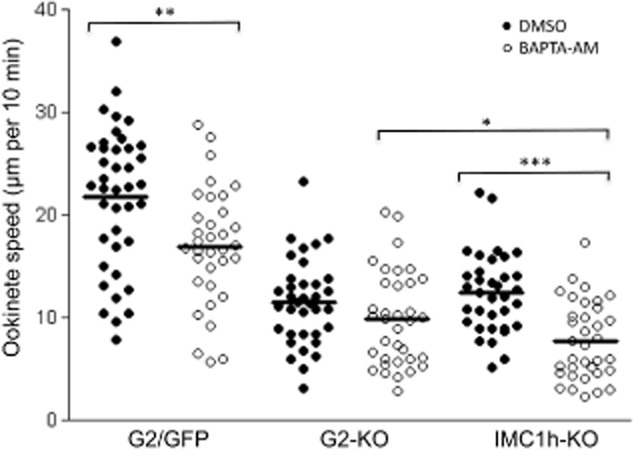
Scatter plot of ookinete motility through matrigel of parasite lines G2/GFP, G2-KO and IMC1h-KO in the absence (closed circles, DMSO solvent control) or presence (open circles) of 10 μM BAPTA-AM. Horizontal lines mark mean values. The plot represents pooled data from two independent experiments (*n* = 35–40). Asterisks indicate statistically significant differences: *P* < 0.05 (*), *P* < 0.001 (**) and *P* < 0.0001 (***) in Mann–Whitney *U*-tests.

The loss of infectivity of the G2 null mutants was unlikely to be caused by a loss of tensile strength. We therefore assessed whether their gliding motility was reduced, as is the case with alveolin IMC1a, IMC1b and IMC1h null mutants. Indeed, the gliding motility of G2-KO ookinetes through matrigel was markedly reduced compared with G2/GFP ookinetes expressing functional G2 (Fig. 6). Over a period of 10 min, G2/GFP ookinetes moved a mean distance of 21.7 μm, while G2-KO ookinetes travelled a mean distance of 11.5 μm (*n* = 40). Accordingly, in this assay gliding motility of the G2-KO ookinetes was only half as efficient as that of its counterparts expressing functional G2. These results show that G2 is not essential for ookinete gliding motility *in vitro*, but that its disruption does adversely affect the ability of the ookinetes to glide normally. In this assay the gliding speed of G2-KO ookinetes was comparable to that of the similarly shaped ookinetes of parasite line IMC1h-KO, in which the alveolin IMC1h is disrupted (Tremp and Dessens, 2011) (Fig. 6). The manner of gliding through matrigel of G2-KO ookinetes (Video S2) was comparable to that of IMC1h-KO ookinetes (Video S3) and displayed a mild meandering pattern, which was less pronounced than in normal-shaped G2/GFP control ookinetes (Video S1). The same was reported independently for IMC1h null mutant ookinetes (Volkmann *et al*., 2012). The decreased meandering could be a result of the reduced cell length and otherwise abnormal shape of these mutant ookinetes, or could reflect a more fundamental difference in gliding motility behaviour.

It is known that intracellular calcium has an important function in apicomplexan zoite motility (Lovett *et al*., 2002; Lovett and Sibley, 2003; Wetzel *et al*., 2004; Ishino *et al*., 2006; Siden-Kiamos *et al*., 2006) and given that G2 possesses a putative calcium-binding domain we tested whether the contribution of this protein to gliding motility was calcium-responsive. If this were the case, it would be expected that motility of G2-KO ookinetes be less affected by reduced intracellular calcium levels than that of ookinetes expressing functional G2. To test this, we assessed ookinete motility in the presence of the intracellular calcium chelator BAPTA-AM, which has previously been shown to reduce ookinete motility through matrigel (Ishino *et al*., 2006). Intracellular calcium also affects other processes involved in motility, for example the calcium-dependent protein kinase 3 (Ishino *et al*., 2006; Siden-Kiamos *et al*., 2006), and an effect of calcium depletion on these processes could mask any effect on G2 function. To minimize this, a concentration of BAPTA-AM was chosen that produced only a relatively small, albeit significant reduction in motility. The presence of 10 μM of the chelator in the matrigel significantly reduced the motility of ookinetes of parasite line G2/GFP (*P* < 0.001) (Fig. 6). Likewise, addition of 10 μM BAPTA-AM significantly reduced motility of IMC1h-KO ookinetes (*P* < 0.0001) (Fig. 6), showing that this effect occurs independently from ookinete shape and their initial gliding speed. In contrast, the same treatment reproducibly resulted in a small and non-significant reduction in motility of G2-KO ookinetes (Fig. 6). Calcium depletion thus appears to adversely affect the function of G2 in cell locomotion, because it reduces motility in G2-positive, but not in G2-negative ookinetes. Thus, the contribution of G2 to ookinete gliding motility appears to be at least in part calcium-responsive and independent of cell shape.

## Discussion

In this study we describe and functionally characterize a new malaria protein named G2, which is predominantly expressed in *P. berghei* ookinetes and sporozoites. G2 and its orthologues show no discernible homology to any proteins with previously defined functions, and hence this study contributes towards the considerable task of characterizing the large number of ‘unknown’ proteins encoded by the parasite. We show that G2 both colocalizes and to a large extend co-purifies with alveolins, suggesting that it is a novel SPN component (Figs 2 and 5). We show that G2 has a predicted N-myristoylation site as well as a calcium binding site, and provide experimental evidence that the protein is myristoylated and calcium-responsive. Many proteins that possess both an N-myristoylation site and a directly downstream-situated calcium binding site posses a calcium myristoyl switch (Lim *et al*., 2011). In such proteins, Ca^2+^-binding causes a conformational change that exposes the myristate group, making the protein more hydrophobic/lipophilic. Accordingly, in biochemical assays the solubility of such proteins is affected by calcium (Lim *et al*., 2011). The fact that calcium depletion reduces deoxycholate-solubility of G2 could therefore indicate that this protein, too, possesses a calcium myristoyl switch, providing it with a potential mechanism for reversible membrane binding.

Besides the pellicle, G2 is also found at the apical end of mature ookinetes in the form of a cap-like structure that contains a pore in the centre. Based on its shape, its position in the cell, its exclusivity to the ookinete and its time of formation, this cap-like structure most likely corresponds to a structure termed ‘collar’. The collar is a distinct electron-dense structure that is laid down towards the end of ookinete development and is situated between the IMC and the subpellicular microtubules in the so-called pellicular cavity at the apical end of the ookinete (Garnham *et al*., 1969; Sinden *et al*., 1985). There is no evidence that the ookinete collar is a membrane-bound compartment/alveolar vesicle like the apical cap in *Toxoplasma*. The collar is one of the last ookinete-specific structures to disappear after oocyst transition (Garnham *et al*., 1969), which also fits well with our observations. Other proteins associated with apical ‘cap-like’ structures have been identified in *T. gondii*: IMC subcompartment protein 1 (ISP1) (Beck *et al*., 2010), and photosensitized INA-labelled protein 1 (PhIL1) (Gilk *et al*., 2006), both of which have orthologues in *Plasmodium*. PhIL1 is associated with the pellicle cytoskeleton and while it localizes to the entire parasite periphery, it is concentrated at the posterior and, particularly, the anterior end basal to the conoid (Gilk *et al*., 2006). In contrast, ISP1 appears associated with the IMC and not the SPN, and is only observed at its apical portion (Beck *et al*., 2010). ISP1 is predicted to be both myristoylated and palmitoylated, but myristoylation or palmitoylation on their own were insufficient to target the protein to the pellicle. Instead, single-acylated ISP1 localized to intracellular membranous structures probably corresponding to the ER and Golgi (Beck *et al*., 2010). These observations fit well with the localization of myristoylated GFP expressed in our G2 null mutants (Fig. 5), as well as in mammalian cells (McCabe and Berthiaume, 1999). It cannot be ruled out that there may be secondary lipid modifications of G2, too, as it possesses numerous predicted palmitoylation sites. Dual acylation has also been reported in the glideosome-associated protein 45 (Rees-Channer *et al*., 2006; Frenal *et al*., 2010). A likely function for these lipid modifications is that they serve to anchor the recipient proteins to the membranes of the IMC.

It is unlikely that G2 fulfils a similar function to the alveolins: G2 does not share any structural homology with the alveolins, nor has it a prominent role in providing tensile strength. In addition, *Plasmodium* alveolins comprise a family of at least 10 distinct molecules that display a substantial degree of differential expression allowing the parasite to ‘manipulate’ its needs for tensile strength by expressing a diverse repertoire of family members in each zoite stage. It is difficult to see why the parasite would require a structurally unrelated protein to add to this already flexible system with regards to providing cell strength. Thus, the independent requirement of both G2 and alveolin expression for normal morphogenesis indicates that zoite cell shape and tensile strength are in fact unlinked. We note that this concept both explains, and is fully supported by, previous observations that the simultaneous knockout of alveolins IMC1b and IMC1h further decreased tensile strength of ookinetes (compared with single knockouts) without further affecting their morphology (Tremp and Dessens, 2011).

One likely cause that is contributing to the reduced infectivity of G2 null mutant ookinetes and sporozoites is reduced gliding motility, as normal gliding motility has been shown to be important for the *in vivo* infectivity of ookinetes (Dessens *et al*., 1999; Yuda *et al*., 1999; Templeton *et al*., 2000) and sporozoites (Sultan *et al*., 1997; Munter *et al*., 2009). Nonetheless, sporozoites that can move only very slowly due to knockout of Hsp20 can still invade hepatocytes *in vivo* at the same efficiency as wild-type sporozoites after intravenous injection, indicating that at least rapid gliding is not essential for efficient invasion (Montagna *et al*., 2012). Moreover, knockout of actin in *T. gondii* indicates that gliding and invasion are at least to a degree uncoupled (Andenmatten *et al*., 2013). The reductions in infectivity of the G2-KO ookinetes and sporozoites may thus not be solely attributable to a reduction in motility. The effect of G2 knockout on motility is likely to occur on several levels. First, the effect on cell shape could adversely affect motility, because a shorter and wider cell is likely to be less efficient at gliding. The same is true for knockouts of the alveolins IMC1a, IMC1b and IMC1h (Khater *et al*., 2004; Tremp *et al*., 2008; Tremp and Dessens, 2011). Second, the effect of calcium depletion on motility of IMC1h-KO ookinetes, but not on G2-KO ookinetes, indicates that G2 plays a role in motility in its own right and independent of cell shape. Third, it is conceivable that an abnormal arrangement of the subpellicular microtubules could be linked to the observed defects in motility in the G2 null mutants. The circular and helical gliding behaviour of crescent-shaped zoites such as *Toxoplasma* tachyzoites and *Plasmodium* ookinetes (Frixione *et al*., 1996; Hakansson *et al*., 1999; Vlachou *et al*., 2004) is thought to be facilitated by the slightly corkscrew fashion in which the subpellicular microtubules run down from the apical rings along the cytosolic face of the pellicle. Moreover, the fixed inclination of the polar rings combined with the arrangement of the subpellicular microtubules have been postulated to provide the *Plasmodium* sporozoite with a dorso-ventral polarity, allowing secretion to occur towards the substrate and cell movement in one preferred direction (Kudryashev *et al*., 2012).

Alveolins also have a role in motility in their own right and independent of cell shape, and are thought to contribute to motility through an association with the glideosome/molecular motor via IMC-resident GAPM transmembrane proteins (Bullen *et al*., 2009; Tremp and Dessens, 2011). Our data suggest that G2 has a role in motility that is calcium-responsive and hence is distinct from that of the alveolins, or at least IMC1h. It has been shown that *T. gondii* tachyzoites exhibit remarkable oscillating intracellular calcium levels during gliding motility (Lovett and Sibley, 2003). Moreover, the frequency of these oscillations is correlated with the gliding speed of the parasites (Lovett and Sibley, 2003). These observations suggest that fluctuations in intracellular calcium are vital for gliding locomotion, but it remains poorly understood what the underlying molecular mechanisms are. Given the properties of G2 demonstrated in this study, the molecule could function to connect the SPN and the IMC through its myristate group. Our findings furthermore show that this interaction could be responsive to intracellular calcium levels. The molecular motor of apicomplexan zoites is tethered to the SPN, and one hypothesis is that fluctuations in intracellular calcium could facilitate traction and motility of the cell through a process of co-ordinated engagement and disengagement of the molecular motor from the SPN. In contrast to ookinetes and sporozoites, *Plasmodium* merozoites only require locomotion for invasion and not to cover distance, which is why they do not possess the more elaborate ‘gliding’ motility of their counterparts in the mosquito. In the context of a role for G2 in gliding motility, it is perhaps not a coincidence that G2 expression is restricted to the ookinete and sporozoite stages. Further studies are underway to test these hypotheses.

Knockout of G2 expression leads to an abnormal morphology of both the ookinete and sporozoite, demonstrating that it is an important molecule for maintaining the cellular architecture. We show for the first time that the cell shape defect of G2 null mutant sporozoites is accompanied by an abnormal organization of the subpellicular microtubules. The same could be true in ookinetes, but due to the large number of microtubules (*c*. 60) and the much larger size of the ookinete we could not establish this. The subpellicular microtubules of *Plasmodium* and related apicomplexa have long been implicated in cell shape and mechanical strength by virtue of being cortical cytoskeletal structures (Morrissette and Sibley, 2002b; Cyrklaff *et al*., 2007). The subpellicular microtubules and the major cell organelles appear to be connected to the IMC via regularly spaced intramembranous particles (Morrissette *et al*., 1997; Kudryashev *et al*., 2010) and G2 could play a role in these putative connections, although a similar periodic distribution of G2 is not apparent. Moreover, the SPN in merozoites has been shown to be thinner than that of sporozoites (Kudryashev *et al*., 2010), which could reflect the absence of G2 expression in the blood-stage zoites. It is difficult to know if the abnormal distribution of the subpellicular microtubules is the cause or the effect of the morphogenesis defects. What is clear is that the morphological abnormalities of the G2-KO zoites are remarkably similar to those observed in alveolin IMC1a, IMC1b and IMC1h knockouts, which suggests that there is a common link between these molecules with respect to zoite morphogenesis. A direct interaction between these molecules cannot be ruled out given their similar temporal and spatial expression patterns. Zoite morphogenesis is concurrent with, and arguably driven by, the simultaneous formation of the IMC and SPN structures (Hu *et al*., 2002; Morrissette and Sibley, 2002a; Tremp *et al*., 2008). It is conceivable that this is a highly constrained molecular process that relies on a finely tuned interaction between these two structures. G2 and alveolins have in common that they are involved in connecting the SPN and IMC, albeit via different mechanisms, and hence knockout of these genes could disturb the SPN-IMC interaction resulting in the irregular zoite morphogenesis observed. This concept would be supported by recent findings that knockout of another SPN-resident protein in *T. gondii*, PhIL1 also alters zoite morphology, making them shorter and wider (Barkhuff *et al*., 2011).

## Experimental procedures

### Parasite maintenance, culture and purification

*Plasmodium berghei* ANKA clone 234 parasites were maintained as cryopreserved stabilates or by mechanical blood passage and regular mosquito transmission. Clone 233, a non-gametocyte producer, was used for preparing asexual blood stages. To purify parasites for genomic DNA extraction, white blood cells were removed from parasitaemic blood by passage through CF11 columns. Ookinete cultures were set up overnight from unpurified gametocytemic blood as previously described (Arai *et al*., 2001). After 18–20 h, ookinetes were purified via ice-cold 0.17 M ammonium chloride lysis and centrifugation at 800 *g* for 10 min, followed by PBS washes. Mosquito transmission assays were as previously described using *An. stephensi* (Dessens *et al*., 1999; 2001; 2003[Bibr b14],[Bibr b15],[Bibr b16]; Le Chat *et al*., 2007).

### Construction of transfection plasmids

The coding sequence of *g2* plus approximately 0.6 kb of the 5′UTR were PCR-amplified with primers pDNR-G2-F (ACGAAGTTATCAGTCGACGGTACCATTTTTGGCTAGATTTTATGACTTA) and pDNR-G2-R (ATGAGGGCCCCTAAGCTTATACATAATGAATATTCTTTTTTTTGCC) and introduced into SalI/HindIII-digested pDNR-EGFP (Carter *et al*., 2008) via In-Fusion cloning to give plasmid pDNR-G2/EGFP. An approximately 0.7 kb sequence corresponding to the 3′UTR of *g2* was PCR-amplified with primers pLP-G2-F (TAAACCATTGGTCATAATGTGATGTCTTTCATATGATTCTC) and pLP-G2-R (CGGCCGCTCTAGCATGAAAAGCATATGATGTTATGAAGATG) and introduced into NdeI-digested pLP-hDHFR2 (Saeed *et al*., 2010) via In-Fusion cloning to give plasmid pLP-DHFR/G2. The *g2*-specific sequence from pDNR-G2/EGFP was introduced into pLP-DHFR/G2 via Cre-*loxP* site-specific recombination to give the final transfection construct pLP-G2/EGFP, used to introduce a GFP-tagged version of *g2* into its locus.

Plasmid pDNR-G2/EFGP served as a template for PCR using primers deltaG2-F (ATTGAAAAAAGCTTAGGGGCCCTCAT) and deltaG2-R (CTAAGCTTTTTTCAATTTCTTCAGACTTTGATATATTT). The amplified plasmid DNA was recircularized via In-Fusion cloning, resulting in the transfection construct pDNR-ΔG2/EGFP, in which all but the first 13 amino acids of the *g2* coding sequence have been removed. The *g2*-specific sequence from pDNR-ΔG2/EGFP was introduced into pLP-DHFR/G2 via Cre-*loxP* site-specific recombination to give the final transfection construct pLP-ΔG2/EGFP. This plasmid was used to introduce a GFP reporter gene into the *g2* locus.

### Generation and genomic analysis of genetically modified parasites

Parasite transfection, pyrimethamine selection and dilution cloning were performed as previously described (Waters *et al*., 1997). Prior to performing transfections, plasmid DNA was digested with KpnI and SacII to remove the vector backbone. Genomic DNA extraction and Southern blot were performed as previously described (Dessens *et al*., 1999). All clonal genetically modified parasite populations were checked for the absence of wild-type parasites by diagnostic PCR.

### Deoxycholate-solubility assays

Purified ookinetes were pelleted by centrifugation, resuspended in extraction buffer [1% deoxycholate, 1% protease inhibitor cocktail (Sigma) in 10 mM Tris-Cl pH 7.5] and incubated on ice for 30 min, followed by centrifugation for 10 min at 16 000 *g* to separate the soluble (supernatant) and insoluble (pellet) protein fractions. Equivalent amounts of each fraction were analysed by Western blot.

### Western blot analysis

Parasite samples were heated in SDS-PAGE loading buffer at 70°C for 10 min. Proteins were fractionated by electrophoresis through NuPage 4–12% Bis-Tris precast gels (Invitrogen) and transferred to PVDF membrane (Invitrogen) according to the manufacturer's instructions. Membranes were blocked for non-specific binding in PBS supplemented with 0.1% Tween 20 and 5% skimmed milk for 1h at room temperature. To detect G2::GFP, goat polyclonal antibody to GFP conjugated to horseradish peroxidase (Abcam ab6663) diluted 1:5000 was applied to the membrane for 1 h at room temperature. To detect CTRP, monoclonal antibody 3A5 (Dessens *et al*., 1999) was used in conjugation with horseradish peroxidase-conjugated goat anti-mouse IgG (Sigma A4416, diluted 1:5000). After washing, signal was detected by chemiluminescence (Pierce ECL Western blotting substrate) according to manufacturer's instructions. Signals were quantified using ImageJ analysis as recommended (http://rsbweb.nih.gov/ij/).

### Tensile strength and viability assays

Unpurified ookinetes present in ookinete cultures were subjected to hypo-osmotic shock of 0.5× normal osmotic strength by adding an equal volume of water. After 5 min, normal osmotic conditions were restored by adding an appropriate amount of 10× PBS. Cell viability was scored by fluorescence microscopy in the presence of 5 ml l^−1^ propidium iodide and 1% Hoechst 33258. Ookinetes whose nucleus stained positive for both propidium iodide and Hoechst were scored as non-viable, whereas ookinetes whose nucleus only stained positive for Hoechst were scored as viable.

### Assessment of ookinete shape and motility

Images of Giemsa-stained ookinetes were captured by microscopy and their length and width measured. The ookinete motility assay was performed as previously described (Moon *et al*., 2009). Ookinete cultures were added to an equal volume of Matrigel (BD Biosciences) on ice in the presence of either 10 μM 5,5′-dimethyl-BAPTA-AM (Sigma) dissolved in DMSO, or an equal volume of DMSO solvent, mixed thoroughly, spotted onto a microscope slide and covered with a Vaseline-rimmed coverslip. The Matrigel was allowed to set at room temperature for at 30 min. Time-lapse videos (1 frame every 10 s for 10 min) were taken on a Zeiss Axioplan II microscope. Movies were analysed with ImageJ using the Manual Tracking plugin (http://fiji.sc/wiki/index.php/Manual_Tracking).

### Microscopy

For assessment of fluorescence, live or paraformaldehyde-fixed parasite samples were assessed, and images captured, on a Zeiss LSM510 confocal microscope. Immunogold labelling was carried out as described (McDonald *et al*., 1995) using rabbit polyclonal antibody to GFP (Abcam, ab6556) diluted 1:500 and goat anti-rabbit IgG 10 nm gold-conjugated (BB International) diluted 1:400. Samples were examined on a Jeol 1200EX Mark II transmission electron microscope and digital images recorded with a 1K 1.3M pixel High Sensitivity AMT Advantage ER-150 CCD camera system.
