# Supplementary material for: Morphogenesis of Plasmodium zoites is uncoupled from tensile strength
Source: Mol Microbiol. 2013 Jul 5;89(3):552–64. doi: 10.1111/mmi.12297 (PMC3912903; doi:10.1111/mmi.12297)

Fig. S1. Sequence and structure of apicomplexan G2. Multiple amino acid sequence alignment of the predicted G2 proteins from *P. berghei* (Pb) and *P. falciparum* (Pf), as well as orthologues found in *Toxoplasma gondii* (Tg), *Cryptosporidium parvum* (Cp), *Theileria annulata* (Ta) and *Babesia bovis* (Bb). The length in amino acids of each of the proteins is indicated by the number at the end. Indicated are residues conserved among at least four of the aligned proteins (shaded), and gaps introduced to allow optimal alignment (hyphens). The alignment was generated with ClustalW.


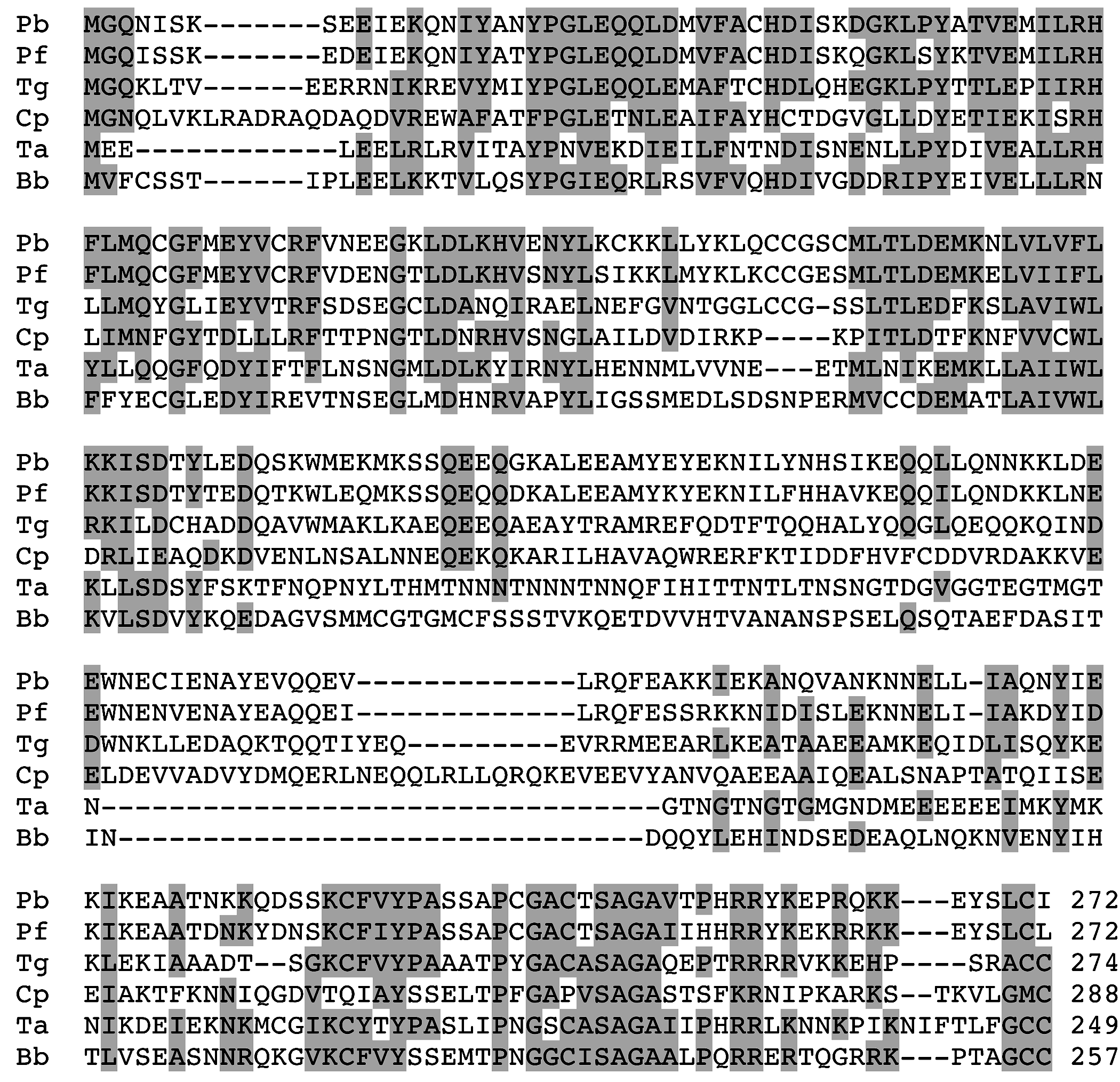

Supplement: Supplementary file 1 [file mmi0089-0552-sd1.docx]
